# A highly-simplified and inexpensive MALDI-TOF mass spectrometry sample-preparation method with broad applicability to microorganisms, plants, and insects

**DOI:** 10.14440/jbm.2018.261

**Published:** 2018-11-22

**Authors:** Michael A. Reeve, Alan G. Buddie, Kathryn M. Pollard, Sonal Varia, Marion K. Seier, Lisa C. Offord, Matthew J.W. Cock

**Affiliations:** CABI, Bakeham Lane, Egham, Surrey, TW20 9 TY, UK

**Keywords:** reagent optimization, sample-preparation, species discrimination, subspecies discrimination

## Abstract

Matrix-assisted laser-desorption and ionization time-of-flight mass spectrometry prepares proteins intact in the gas phase with predominantly a single positive charge. The times-of-flight of charged proteins along a tube held at high vacuum after acceleration in an electrical field are proportional to the square root of the mass-over-charge ratios for the proteins, thereby allowing a mass spectrum to be generated, which can then be used to characterize or identify a protein-containing sample. Several sample-preparation methods are currently available but not all of these are applicable to some forms of fungal biomass and few of these are well suited to the analysis of plant or insect material. We have therefore developed a simplified method that: lyses cells, selectively solubilizes basic proteins, dissolves matrix to a suitable concentration, generates spectra with good intensity and peak richness, costs no more (and generally less) than current methods, and is not constrained in terms of throughput by the availability of centrifuges. Using this method, and a reagent formulation comprising α-cyano-4-hydroxycinnamic acid matrix close to saturation in 60%–65% (v/v) acetonitrile in water containing 2.5% (v/v) trifluoroacetic acid, we have been able to differentiate between strains for a representative subset of aflatoxin-producing and aflatoxin-non-producing strains of *Aspergillus* fungi, to differentiate between Indian and Pakistani strains of Himalayan balsam rust, to differentiate between closely-related *Crassula* spp. and regional biotypes of *Crassula helmsii*, and to differentiate between rubbervine introduced into Australia and Brazil. We have also analyzed fall armyworm and stem-borer samples stored in 70% (v/v) ethanol and old dried insect specimens.

## INTRODUCTION

### MALDI-TOF MS and MALDI-TOF MS sample-preparation

Matrix-assisted laser-desorption and ionization time-of-flight mass spectrometry (MALDI-TOF MS) employs MALDI soft ionization of biological samples [1]. In this process, large proteins can be prepared intact in the gas phase with predominantly a single positive charge [2]. The time-of-flight of a charged protein along a tube held at high vacuum after acceleration in an electrical field is proportional to the square root of the mass-over-charge ratio for the protein, thereby allowing a mass spectrum to be generated from the time-of-flight values for the protein components in a particular biological sample. The mass spectrum of a subset of the expressed proteome of a biological sample (normally the highly-expressed acid-soluble proteins, including many ribosomal proteins) is a versatile and sensitive tool for the characterization and identification of the sample.

Several MALDI-TOF MS sample-preparation methods are currently available [3-8]. For the identification of bacteria and yeasts, “direct-transfer” protocols (also known as “intact-cell” protocols) are generally used. In these methods, material from a single microbial colony from an agar plate is first smeared on a sample-plate target zone (normally using a sterile toothpick). This is then overlaid with 1 μl of matrix (often α-cyano-4-hydroxycinnamic acid (HCCA) in 50% (v/v) aqueous acetonitrile containing 2.5% (v/v) trifluoroacetic acid (TFA)), followed by drying and loading into the mass spectrometer. In some protocols, an additional overlay of 1 μl of 70% (v/v) aqueous formic acid followed by drying is carried out prior to the overlay of matrix. For filamentous fungal identification, most researchers currently follow a ‘full-extraction’ protocol based on that of Cassagne *et al*. [9]. In the original method, fungi are incubated on Sabouraud-gentamicin-chloramphenicol plates for 72 h at 27°C. Fungal material is scraped from the colony and placed in 300 μl of water. 900 μl of ethanol are then added, followed by centrifugation for 10 min at 13000 rpm in an Eppendorf centrifuge. The pellet is incubated for five minutes in 10 μl of 70% (v/v) aqueous formic acid. Ten microliters of acetonitrile are then added, followed by centrifugation for two minutes at 13000 rpm. One microliter of the supernatant is then pipetted onto the sample-plate target zone and dried. This is finally overlaid with 1 μl of matrix, followed by drying and loading into the mass spectrometer.

Whilst direct-transfer protocols are rapid to perform, they are not well suited to the transfer of some types of fungal biomass, especially powdery forms of mycelium and spores. Full-extraction protocols are easier in this regard but are vulnerable to loss of biomass after the first centrifugation step when small amounts of material are used. In addition, throughput can be limited by the centrifugation steps. Neither method is particularly convenient for the analysis of plant material [10] or insect material [11] and, in the case of plant material, concerns have been expressed about the potential for high levels of ribulose bisphosphate carboxylase-oxidase (RUBISCO) proteins to interfere with MALDI-TOF MS-based analysis [10]. Longer term, it would therefore be desirable to develop methods for filamentous fungal identification (and also the identification of other biological materials such as plants, insects, nematodes, and oomycetes) that do not require repeated and time-consuming centrifugation, thereby making MALDI-TOF MS-based analysis even better suited to the diagnosis of crop disease in developing countries, which is a particular aim of this work. In order to simplify MALDI-TOF MS sample-preparation as much as possible, a single reagent would ideally be developed which would: lyse cells, selectively-solubilize ribosomal (and other acid-soluble) proteins, dissolve HCCA to sufficiently high concentration, generate spectra of suitable intensity and peak richness, and cost no more (and preferably less) than current methods. Sample-preparation would then simply be a matter of adding biomass to such a solution followed by pipetting 1 μl of the resulting cell lysate onto the sample-plate target zone, drying, and loading into the mass spectrometer. In the current paper, we have developed such a simplified method and demonstrate its potential value for the analysis of microorganisms, plants, and insects.

To test the simplified method developed for MALDI-TOF MS sample-preparation, we employed materials that were readily available at the time, which would broadly test the applicability of the method, and which might form the basis for preliminary studies to assess the suitability of MALDI-TOF MS as an analytical tool to solve existing problems in microbiology, the biological control of invasive plant species, and the identification of insect species. For the six examples that we selected, introductory background material is presented below.

### Strains for a representative subset of aflatoxin-producing *vs*. aflatoxin-non-producing strains of *Aspergillus* fungi

Mycotoxins are secondary metabolites of various chemical classes produced by a broad range of fungi. Their presence in commodities can be deleterious to the health of humans and/or animals. As a consequence, these compounds are becoming increasingly important in food security, and several are also subject to regulatory requirements. Probably the most significant mycotoxins are the aflatoxins: a group of heterocyclic, oxygen-containing compounds with a bisdifurano ring system [12]. Several classes of aflatoxin have been described, of which B1 and B2 (collectively AFB) and G1 and G2 (collectively AFG) are the most significant fungal products. It is believed that *Aspergillus flavus* strains often produce AFB toxins, whilst some isolates of *Aspergillus parasiticus* are able to produce AFB and AFG [13]. These compounds have been shown to be toxic to humans and animals, with AFB1 in particular listed as carcinogenic to humans by the International Agency for Research on Cancer [14]. *A*. *flavus*, *A*. *parasiticus*, and others which may also produce aflatoxins belong to *Aspergillus* section *Flavi*. The situation is however more complex because some *A*. *flavus* isolates have been shown to be incapable of producing any aflatoxins. Such atoxigenic strains have been demonstrated to have considerable potential in controlling aflatoxin levels by out-competing toxin-producing strains in the field [15].

Given the importance of minimizing aflatoxin levels in crop commodities, it is not surprising that researchers have already turned to MALDI-TOF-MS to attempt to resolve species and/or toxin production within *Aspergillus* section *Flavi* [13,16]. *Aspergillus* section *Flavi* contains 25 species, of which six species are significant economically. These are the toxin-producing species *A*. *flavus*, *A*. *parasiticus*, and *A*. *nomius* along with the non-toxigenic species *A*. *oryzae*, *A*. *sojae*, and *A*. *tamarii*, which are used in food production. Methods for discriminating between these species have been investigated previously [16]. Whilst researchers are agreed on the capability of MALDI-TOF MS to resolve at the species level, there has been some disagreement on the method’s ability to discriminate between toxigenic strains and those that are non-toxigenic. Li and co-workers [17] claimed that they could differentiate between strains by MALDI-TOF-MS on the basis of their toxin-production ability but this has since been disputed by da Silva and co-workers [16], who were unable to do so with their particular strains and methodology. Moreover, the latter study showed considerable similarity between members of *A*. *flavus* and *A*. *oryzae*, leading the authors to propose synonymizing the species—a view which is now generally accepted by the mycology community. With the above in mind, we chose to analyze a representative subset of aflatoxin-producing and aflatoxin-non-producing strains of *Aspergillus* fungi using our simplified MALDI-TOF MS method.

### India-strain *vs*. Pakistan-strain Himalayan balsam rust (*Puccinia komarovii* var. *glanduliferae*)

Himalayan balsam (*Impatiens glandulifera*) is an annual plant native to the foothills of the Himalayas which was introduced into the UK in 1839 for its ornamental purposes and has since spread prolifically. It can form dense monospecific stands which can out-compete native vegetation, decrease biodiversity, and increase flood risk and erosion [18-20]. In 2006, CABI initiated a biological control programme against Himalayan balsam and conducted natural enemy surveys across the native range. A rust fungus, identified as *Puccinia komarovii* var. *glanduliferae* [21] was prioritized due to its prevalence and high level of damage in the field. In 2014, after years of rigorous safety testing and governmental approval, a strain of the rust fungus from the Kullu Valley, Himachal Pradesh, India (IMI 398718) was released in the UK as a biological control agent [22]. The rust fungus was released at field sites across England and Wales in 2015 and 2016 and results suggested that distinct biotypes of Himalayan balsam exist, with some populations being susceptible to infection whilst others were resistant [23]. As rust fungi are highly host-specific, it is believed that different strains of the rust exist which have evolved with distinct biotypes or populations of the plant. In response to this hypothesis, a strain from Kaghan Valley, Khyber Pakhtunkhwa Province, Pakistan (IMI 505791) was retrieved from liquid nitrogen and its host-range confirmed through testing of a number of closely-related species [23]. Initial assessments found that the strain from Pakistan can infect some of the Himalayan balsam populations that were resistant to the Indian strain. Permission to release this strain was granted in January 2017 and, after conducting susceptibility assessments for each population, the most virulent strain of the rust was released at field sites during 2017. At some sites, both the Indian and Pakistani strains were released. In order to determine which strain performs better under given field conditions, it would be highly desirable to develop a method that could distinguish between spores derived from these two rust strains. MALDI-TOF MS proteotyping using our simplified method was therefore tested as a possible basis for such discrimination.

### Closely-related *Crassula* spp. *vs*. regional biotypes of *Crassula helmsii*

*Crassula helmsii* or Australian swamp stonecrop is a semi-aquatic plant species native to Australia and New Zealand that was introduced to Europe in the early 20^th^ century as a pond plant [24]. Growing in still, and slow-moving, water bodies, *C*. *helmsii* can dominate ecologically-sensitive habitats, can cause operational problems for the water industry, and can affect leisure activities. *C*. *helmsii* is now widespread in the UK and is localized in other parts of Western Europe and, as a result, CABI has been investigating the classical biological control of this species in the UK using the gall-forming Australian mite, *Aculus* sp. (Eriophyidae). Mites from this family are renowned for their high host-specificity (even to plant biotype) and gall-forming species from this family can be particularly specific [25]. Understanding the extent of variation within the target weed population prior to the release of the biological control agent is therefore a crucial step to ensure that plant populations are susceptible to the agent under investigation. As C. *helmsii* mainly grows vegetatively, it has been assumed that all plant populations in the UK will be fairly homogeneous. Recent evidence has, however, shown that these plants can also produce viable seeds [26] and, in addition, it is not clear how many introductions there have been into the UK from the native range. As a result, it would be of considerable benefit if we were able rapidly and inexpensively to discriminate between different populations of C. *helmsii*, with the aim of matching the best mite biotype with a particular plant biotype. MALDI-TOF MS proteotyping offers one such possibility and therefore closely-related *Crassula* spp. and regional biotypes of Australian swamp stonecrop (*Crassula helmsii*) were analyzed using our simplified method.

### Rubbervine species introduced into Australia *vs*. Brazil

The ability to separate closely-related plant species from each other or individual plant species down to the varietal or even biotype level is crucial when using co-evolved fungal pathogens in classical weed biocontrol. Successful control relies upon a compatible and virulent host-pathogen interaction and thus upon the best match between the plant host and the pathotype of the selected fungal species [27,28]. The Australian biocontrol programme against the invasive Madagascan climber rubbervine (*Cryptostegia grandiflora*) represents an excellent example highlighting the importance of such matching. Following a thorough assessment regarding suitability and safety, the highly-host-specific rust *Maravalia cryptostegiae*, associated with rubbervine in its native range, was approved for import into Australia in 1994 as a classical control agent for this weed [29]. First releases of the rust, however, did not result in the anticipated high levels of infection on the invasive weed [30]. Further investigation revealed that the introduced rust isolate had been mistakenly collected from the closely-related species *Cryptostegia madagascariensis* as, at the time, the distinguishing morphological characteristics of these two *Cryptostegia* species, and their potential to hybridize, were not well understood [31,32]. Subsequent re-collection, import, and release of an isolate of *M*. *cryptostegiae* associated with *C*. *grandiflora* rectified the problem and turned the biocontrol programme into one of the most successful ever undertaken, with a cost-benefit ratio of 1:108 [33,34]. Lessons learned from this initiative will be applied to tackle the invasive devil’s claw (*C*. *madagascariensis*) in north-eastern Brazil, where this species poses a serious threat to the fragile ecosystem of the Caatinga [35]. In order to collect and evaluate the best-matched pathotype of the rust *M*. *cryptostegiae* as a potential classical biocontrol agent for introduction into Brazil, it will be crucial to pinpoint the area of origin of the invasive Brazilian population(s). To achieve this, a reliable and comparatively-cheap method which is able to match dried leaf material collected from a transect of Madagascan *Cryptostegia* field populations with leaf material from invasive populations in Brazil is needed. With this in mind, we assessed the suitability of our simplified MALDI-TOF MS method for this purpose.

### Analysis of fall armyworm and stem-borer samples stored in 70% ethanol

The fall armyworm, *Spodoptera frugiperda* (Lepidoptera, Noctuidae) is native to the tropical regions of North and South America, ranging from the United States to Argentina [36]. It is on the European Plant Protection Organization (EPPO) A1 list of quarantine pests and is a cause of major damage to economically-important cultivated crops [37]. In 2016, fall armyworm was reported for the first time in sub-Saharan Africa [38]. Its spread since has been rapid and, at the time of writing, the presence of fall armyworm has been verified in at least 20 African countries, and it is expected to spread rapidly to all suitable African countries, and beyond [39] in the next year or two. The fall armyworm develops through six instars and, whilst the moths are relatively short-lived compared to the larval forms, they are able to fly long distances using air currents in order to colonize new sites. Whilst identification of adult moths is well documented, visual identification of the larval stages (and discrimination from stem-borers and other types of armyworm) requires entomological experience, and is only practical for later instars (which are characterized by an inverted Y on the face and four spots in a square arrangement on the last segment of the body). The larvae are responsible for the observed crop damage, and later instars have a voracious appetite, feeding on maize, rice, sugarcane, cotton, and vegetables in particular. Two populations of fall armyworm are known [40], referred to as the rice (or type 1) strain (feeding principally on rice and pasture grasses) and the corn (or type 2) strain (feeding principally on corn, cotton, and sorghum), and have been characterized from Africa using DNA barcoding [38,41]. Additional studies have demonstrated that genes associated with host preferences do not always correspond with the strains characterized by their DNA barcodes [42]. Given the economic importance of this invasive pest, we were motivated to investigate whether our simplified method for MALDI-TOF MS analysis could be used to discriminate between larvae of fall armyworm (including their known races characterized by DNA-barcoding) and stem-borers.

### Analysis of old dried insect specimens of Sphingidae (Lepidoptera)

Rapid, reliable, and cheap identification of biological samples is a key aim for diagnostic and identification support to agricultural extension, phytosanitary services, biodiversity monitoring, compliance with trade regulations as well as many research activities involving biological material, particularly in the field [43,44]. The challenges of doing this with insects are particularly great due to their enormous diversity, complex functional roles, the inadequacy of current taxonomic knowledge, and the shortage of trained taxonomists, particularly in less affluent countries [41]. Molecular methods, particularly those using DNA barcoding, are being explored for ways in which they can support and complement taxonomic activities in these areas [45,46]. Reliable reference material for many insects is old and, in the case of unique type material on which species are based, much of this is over 100 years old. Until now, it has been difficult and costly to extract DNA sequences from older material [47], although that may change as methods are further improved [48,49]. In this study, we have used preserved dry specimens of Sphingidae (Lepidoptera) because this family (comprising hawk moths, sphinx moths, and hornworms) is well understood taxonomically, based on morphological and molecular approaches, this information is readily available [50,51], and suitable preserved sample material was immediately available to us. MALDI-TOF MS analysis may provide an alternative and complementary approach to barcoding which, if it works for Sphingidae, may be developed for other insect groups. In order to test this, old dried insect specimens were characterized using our simplified method.

## MATERIALS AND METHODS

### Mass spectrometry

Mass spectrometry covering the range 2 kDa to 20 kDa was carried out using a Bruker Microflex LT linear-mode instrument running the MALDI Biotyper 4.0 applications (Bruker Daltonik, Bremen, Germany), using a nitrogen laser at 337 nm, with 240 laser shots per sample, and an ion-source voltage of 19.98 kV. Calibration was carried out using the manufacturer’s ‘BTS’ controls (*E. coli* proteins supplemented with ribonuclease A and myoglobin), using peaks with masses at 3637.8; 5096.8; 5381.4; 6255.4; 7274.5; 10300.2; 13683.2, and 16952.3 for calibration according to the manufacturer’s instructions. Spectra were acquired using MALDI Biotyper RTC Version 4.0 (Build 19) using the manufacturer’s standard settings (Centroid peak-detection algorithm and TopHat baseline subtraction). Database entries were made as single-spectra MSPs using the Bruker Online Client software suite (Version 4.0.19, Bruker Daltonik, Bremen, Germany) using the manufacturer’s standard settings. For spectral comparisons, Bruker identification scores were derived using the standard Bruker algorithm. This first converts raw mass spectra into peak lists, which are then compared between spectra. Three separate values are computed: the number of peaks in the reference spectrum that have a closely-matching partner in the test spectrum (value range 0–1), the number of peaks in the test spectrum that have a closely-matching partner in the reference spectrum (value range 0–1), and the peak-height symmetry of the matching peaks (value range 0–1). The above three values are multiplied together and normalized to 1000, and the base-10 logarithm is then taken to give the final Bruker score (range 0–3). Bruker scores of scores between 2.3 and 3.0 indicate very close relatedness, scores between 2.0 and 2.3 indicate close relatedness, and scores below 1.7 indicate low relatedness.

### Development of a suitable reagent formulation

≥ 99.8% ethanol, LC-MS-grade water, ≥ 99.0% (HPLC-grade) HCCA matrix, ≥ 98% (TLC-grade) HCCA matrix, LC-MS-grade acetonitrile, and 99% ReagentPlus^®^-grade TFA were purchased from Sigma (Gillingham, UK).

To investigate whether a compatible solvent system can be formulated for combined cell lysis, ribosomal-protein extraction, and matrix dissolution, bacterial cells (*Pseudomomas syringae* IMI 349156) were scraped from colonies grown on nutrient agar and resuspended in water until turbid to OD_600_ ~2. One hundred microliter aliquots were then pelleted by centrifugation at 14100 G for two minutes in a miniSpin^®^ plus centrifuge (Eppendorf, Stevenage, UK). Pellets were resuspended in 20 μl of saturated solutions of HPLC-grade HCCA matrix in 100%, 90%, 80%, 70%, 60%, 50%, 40%, 30%, 20%, and 10% (v/v) acetonitrile in water or 100%, 90%, 80%, 70%, 60%, 50%, 40%, 30%, 20%, and 10% (v/v) acetonitrile in water containing 2.5% (v/v) TFA. Samples were then either centrifuged at 14100 G for two minutes and 1 μl of supernatant was deposited on the MALDI-TOF MS sample plate followed by drying or samples were vortex mixed and 1 μl of the resulting crude lysate was deposited on the MALDI-TOF MS sample-plate followed by drying and analysis. Full-extraction controls were performed according to the method of Cassagne *et al*. [9], as described in the Bruker handbook [6].

For titration of the optimal amount of TFA in the reagent formulation, saturated solutions of HPLC-grade HCCA matrix were made up in various aqueous acetonitrile and TFA mixtures as indicated. Equal amounts of *Pseudomomas syringae* IMI 349156 biomass were then resuspended in 20 μl of each reagent and 1 μl was pipetted onto the sample-plate before drying and analysis.

### Strains for a representative subset of aflatoxin-producing *vs*. aflatoxin-non-producing strains of *Aspergillus* fungi

The chosen strains expected to be aflatoxin-producing were *Aspergillus flavus* (IMI 190443), *Aspergillus flavus* (IMI 242693), *Aspergillus flavus* (IMI 92875ii), *Aspergillus parasiticus* (IMI 120920), *Aspergillus parasiticus* (IMI 89717), and *Aspergillus parasiticus* (IMI 91019b). The chosen strains expected to be aflatoxin-non-producing were *Aspergillus flavus* (IMI 357038), *Aspergillus flavus* (IMI 93803), and *Aspergillus oryzae* (IMI 126842). Aflatoxin-non-producing *Aspergillus tamarii* (IMI 91888) was also chosen as an ‘outgroup’ to act as a positive control for the identification process. Samples of aflatoxin-producing and aflatoxin-non-producing strains of *Aspergillus* fungi were grown on duplicate nine-centimetre distilled-water malt-extract agar (DWMEA) plates for three days at 25°C in the dark. Fungal biomass was mixed with 60 μl of 12 mg/ml TLC-grade HCCA matrix in 60% (v/v) acetonitrile, 2.5% (v/v) TFA, and 37.5% (v/v) water using a plastic inoculating loop. One microliter of the resulting crude lysate was then pipetted onto the Bruker sample-plate, air dried, and loaded into the spectrometer. Full-extraction controls were performed according to the method of Cassagne *et al* [9], as described in the Bruker handbook [6]. Using the Bruker Online Client software suite (version 4.0.19, Bruker Daltonik, Bremen, Germany), two reference samples for each strain (plate 1 and plate 2) were used to construct database mass spectra (as single-spectra MSPs using the Bruker Online Client software), which were then used to identify two test samples (again, plate 1 and plate 2) for each strain. Each test sample was identified (again using the Bruker Online Client software) against all twenty reference spectra (ten strains, plate 1 and plate 2). The resulting logarithmic Bruker scores between 0 and 3 were converted back to linear scores between 0 and 1000. The resulting linearized values for all reference spectra derived from each strain were averaged and the strain with the highest average figure was selected as the identification call (indicated in emboldened font in the results section). The percentage clearance (defined as the percentage gap between the highest average value and the second-highest average value) was then calculated as a crude measure of the identification confidence, with 0%–10% clearance arbitrarily defined as low-confidence, 10%–20% clearance as reasonable confidence, and better than 20% clearance as high confidence.

### India-strain *vs*. Pakistan-strain Himalayan balsam rust (*Puccinia komarovii* var. *glanduliferae*)

Eight samples of Himalayan balsam rust urediniospores (four from India and four from Pakistan) in 70% (v/v) ethanol were analyzed. Fifty microliter aliquots of urediniospores were pelleted by centrifugation for one minute at 14100 G and the pellet was macerated in 50 μl of 12 mg/ml TLC-grade HCCA in 65% (v/v) acetonitrile, 2.5% (v/v) TFA, and 32.5% (v/v) water using the blunt end of a plastic inoculating loop. The tube was centrifuged for 1 min at 14100 G and 1 μl of the resulting supernatant was then pipetted onto the Bruker sample-plate, air dried, and loaded into the spectrometer. Duplicate MALDI-TOF MS sample -preparations were carried out for each sample of rust spores. Duplicate samples from each preparation were pipetted onto the MALDI-TOF MS sample-plate. Spectra were analyzed by principal-component analysis (PCA) using the Bruker Online Client software, with the components of the first principal component vector shown in the *X* direction and the components of the orthogonal second principal component vector shown in the *Y* direction to give an ordination plot. PCA analysis was unsupervised, and all peaks were weighted equally.

### Closely-related *Crassula* spp. *vs.* regional biotypes of *Crassula helmsii*

Six reference leaves and six ‘blind-test’ leaves were analyzed from each of: *Crassula aquatica*; *Crassula helmsii*, UK; *Crassula helmsii*, Victoria, Australia; *Crassula helmsii*, Flinder’s Island, Australia; and *Crassula helmsii*, Belgium. Individual leaves washed in 70% (v/v) ethanol were macerated in 60 μl of 12 mg/ml TLC-grade HCCA in 60% (v/v) acetonitrile, 2.5% (v/v) TFA, and 37.5% (v/v) water using the blunt end of a plastic inoculating loop. One microliter of the resulting crude lysate was then pipetted onto the Bruker sample-plate, air dried, overlaid with a further 1 μl of 12 mg/ml TLC-grade HCCA in 60% (v/v) acetonitrile, 2.5% (v/v) TFA, and 37.5% (v/v) water, air dried, and loaded into the spectrometer. Using the Bruker On-line Client software, reference samples were used to construct database mass spectra, which were then used to identify the unknown ‘blind-test’ samples.

### Rubbervine species introduced into Australia *vs.* Brazil

Pressed-and-dried samples of Australian rubbervine and Brazilian rubbervine were analyzed. Two reference leaf fragments from each of two leaves from each of two plants along with two “blind-test” leaf fragments from each of two leaves from each of two plants were analyzed from each type of rubbervine. Roughly 3 mm × 3 mm fragments of leaf biomass soaked overnight in 70% (v/v) ethanol were macerated in 60 μl of 12 mg/ml TLC-grade HCCA in 60% (v/v) acetonitrile, 2.5% (v/v) TFA, and 37.5% (v/v) water using the blunt end of a plastic inoculating loop. One microliter of the resulting crude lysate was then pipetted onto the Bruker sample-plate, air dried, overlaid with a further 1 μl of 12 mg/ml TLC-grade HCCA in 60% (v/v) acetonitrile, 2.5% (v/v) TFA, and 37.5% (v/v) water, air dried, and loaded into the spectrometer. Using the Bruker On-line Client software, reference samples were used to construct database mass spectra, which were then used to identify the unknown blind-test samples.

### Analysis of fall armyworm and stem-borer samples stored in 70% ethanol

The preliminary study reported was performed blind with six insect samples (PEST 1-6) archived in 70% (v/v) ethanol for up to nine months. Each sample (roughly 1 mm^3^ of insect biomass) was macerated in 100 μl of 11 mg/ml TLC-grade HCCA in 65% (v/v) acetonitrile, 2.5% (v/v) TFA, and 32.5% (v/v) water using the blunt end of a plastic inoculating loop. One microliter of the resulting crude lysate was then pipetted onto the Bruker sample-plate, air dried, and loaded into the spectrometer. Triplicate sample-preparations (A, B, and C) were carried out from each insect and principal-component analysis was then carried out from the A samples using the Bruker Online Client software to generate a PCA dendrogram.

### Analysis of old dried insect specimens of the family Sphingidae

Adult insect legs were taken from the Sphingidae species indicated, that had been held in the research collection of M.J.W. Cock, and which had been stored dry since sampling on the dates indicated. Samples were initially processed blind. Each sample was macerated in 60 μl of 12 mg/ml TLC-grade HCCA in 60% (v/v) acetonitrile, 2.5% (v/v) TFA, and 37.5% (v/v) water using the blunt end of a plastic inoculating loop. One microliter of the resulting crude lysate was then pipetted onto the Bruker sample-plate, air dried, overlaid with a further 1 μl of 12 mg/ml TLC-grade HCCA in 60% (v/v) acetonitrile, 2.5% (v/v) TFA, and 37.5% (v/v) water, air dried, and loaded into the spectrometer. Using the Bruker On-line Client software, a PC1 versus PC2 PCA ordination plot was generated and samples were also used to construct database mass spectra which were then used to cross-identify all samples in order to examine their relatedness. PCA analysis was unsupervised, and all peaks were weighted equally.

## RESULTS

### Development of a suitable reagent formulation

**Figure 1.**
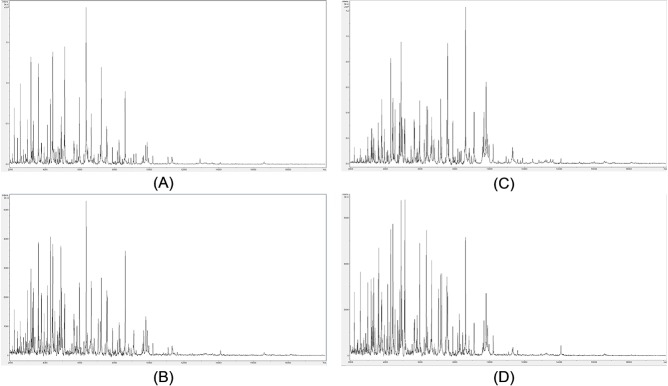
Example *Pseudomomas syringae* (IMI 349156) mass spectra from solvent-optimization titrations. Example mass spectra (baseline-subtracted, smoothed, *y*-axis-autoscaled, and covering the mass range 2 kDa to 20 kDa) from solvent-optimization titrations, with 80% (v/v) acetonitrile, 2.5% (v/v) TFA, and saturated HCCA (A); 70% (v/v) acetonitrile, 2.5% (v/v) TFA, and saturated HCCA (B); 60% (v/v) acetonitrile, 2.5% (v/v) TFA, and saturated HCCA (C); and full-extraction control (D).

To investigate whether a compatible solvent system can be formulated for combined cell lysis, acid-soluble-protein extraction, and matrix dissolution, bacterial cells (*Pseudomomas syringae* IMI 349156) were employed. The concentration of acetonitrile was first optimized by varying the acetonitrile concentration in aqueous solutions containing HCCA at saturation, with and without 2.5% (v/v) TFA. Example spectra are shown in **Figure 1**. Spectra were obtained using between 60% (v/v) acetonitrile and 80% (v/v) acetonitrile (**Fig. 1A-1C**). In the absence of TFA, mass spectra with significantly fewer peaks are obtained (data not shown). In the presence of TFA, mass spectra are obtained with comparable peak richness to full-extraction controls (**Fig. 1D**). Subjective mass spectral quality improves slightly when the acetonitrile concentration is reduced from 80% (v/v) to 60% (v/v) (as does the ease of pipetting onto the sample plate). Reagent formulations employing either 60% (v/v) or 65% (v/v) acetonitrile were therefore used for the examples below.

**Figure 2.**
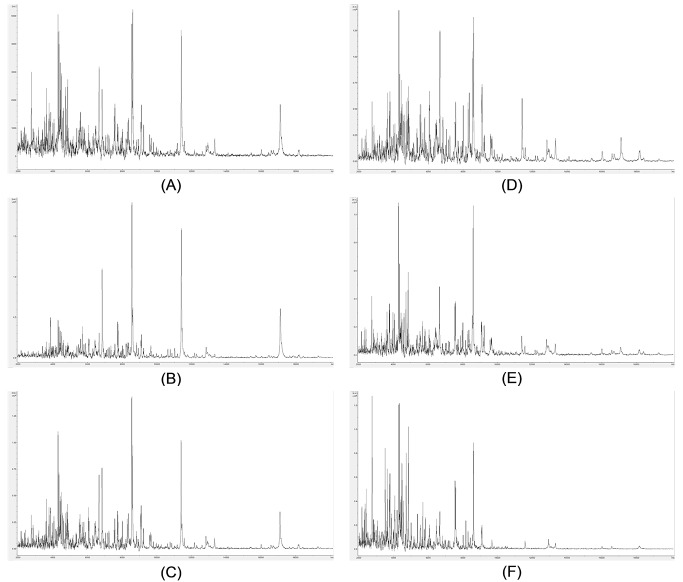
Example *Pseudomomas syringae* (IMI 349156) mass spectra from TFA-optimization titrations. Example mass spectra (baseline-subtracted, smoothed, y-axis-autoscaled, and covering the mass range 2 kDa to 20 kDa) from TFA-optimization titrations, with 65% (v/v) acetonitrile, 10% (v/v) TFA, and saturated HCCA (A); 65% (v/v) acetonitrile, 5% (v/v) TFA, and saturated HCCA (B); 65% (v/v) acetonitrile, 2.5% (v/v) TFA, and saturated HCCA (C); 65% (v/v) acetonitrile, 1.25% (v/v) TFA, and saturated HCCA (D); 65% (v/v) acetonitrile, 0.63% (v/v) TFA, and saturated HCCA (E); and 65% (v/v) acetonitrile, and saturated HCCA (F).

For titration of the optimal amount of TFA, the acetonitrile concentration was set at 65% (v/v), HCCA was again used at saturation, and bacterial biomass (*Pseudomomas syringae* IMI 349156, as above) was employed. Example spectra are shown in **Figure 2**. Bacterial samples give mass spectra from 10% (v/v) TFA (**Fig. 2A**) down to no TFA included (**Fig. 2F**). At lower TFA concentrations, higher molecular weight proteins are progressively lost from the mass spectrum. Two point five percent (v/v) (**Fig. 2C**) is the lowest TFA concentration at which such high molecular weight proteins are still well represented in the mass spectrum and the spectrum for 2.5% (v/v) TFA is slightly more complex than that for 5% (v/v) TFA (**Fig. 2D**). 2.5% (v/v) TFA was therefore used for the examples below.

The optimal reagent formulation for the simplified method was therefore set at HCCA matrix as close to saturation as is practical (determined empirically at between 11 mg/ml and 12 mg/ml) in 60%–65% (v/v) acetonitrile in water containing 2.5% (v/v) TFA.

### Strains for a representative subset of aflatoxin-producing *vs*. aflatoxin-non-producing strains of *Aspergillus* fungi

*Aspergillus flavus* (IMI 190443), *Aspergillus flavus* (IMI 242693), *Aspergillus flavus* (IMI 92875ii), *Aspergillus parasiticus* (IMI 120920), *Aspergillus parasiticus* (IMI 89717), and *Aspergillus parasiticus* (IMI 91019b), which were expected to be aflatoxin-producing and *Aspergillus flavus* (IMI 357038), *Aspergillus flavus* (IMI 93803), *Aspergillus oryzae* (IMI 126842), and *Aspergillus tamarii* (IMI 91888), which were expected to be aflatoxin-non-producing were analyzed by MALDI-TOF MS using the simplified method and the full-extraction protocol. Averaged linearized identification values for test samples against reference samples and percentage-clearance values are given for the simplified protocol and for the full-extraction protocol in **Table S1**. No spectra were obtained for the FLAVUS IMI 357038 test or reference spectra using the full-extraction controls. As can be seen in **Table S1**, all of the aflatoxin-producing and aflatoxin-non-producing strains used in this study can be discriminated using the simplified protocol but one identification error was obtained with the full-extraction controls.

### India-strain *vs*. Pakistan-strain Himalayan balsam rust (*Puccinia komarovii* var. *glanduliferae*)

**Figure 3.**
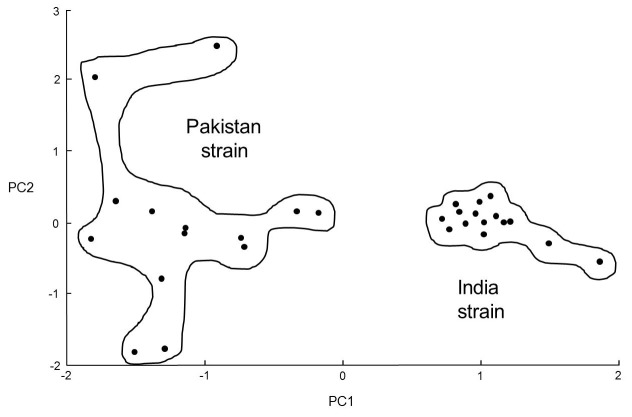
PCA ordination plot for India-strain and Pakistan-strain Himalayan balsam rust spectra. PCA ordination plot for the India-strain and Pakistan-strain Himalayan balsam rust (*Puccinia komarovii* var. *glanduliferae*) urediniospore mass spectra (figure redrawn from original Bruker image). Spectra were analyzed by PCA using the Bruker Online Client software, with the components of the first principal component vector shown in the *X* direction and the components of the orthogonal second principal component vector shown in the *Y* direction to give the ordination plot. PCA analysis was unsupervised, and all peaks were weighted equally.

**Figure 4.**
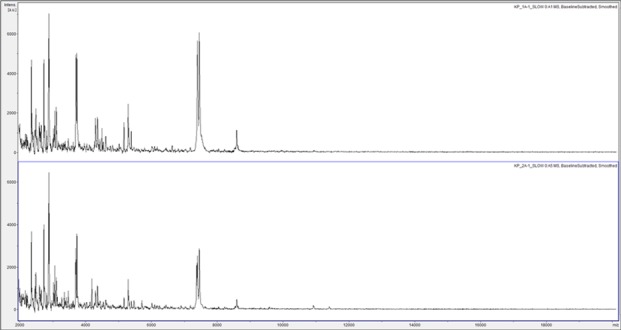
Example mass spectra for India-strain and Pakistan-strain Himalayan balsam rust. Example mass spectra (baseline-subtracted, smoothed, y-axis-autoscaled, and covering the mass range 2 kDa to 20 kDa) for the India-strain Himalayan balsam rust (*Puccinia komarovii* var. *glanduliferae*) (top) and the Pakistan-strain Himalayan balsam rust (bottom).

Eight samples of Himalayan balsam rust urediniospores (four from India and four from Pakistan) were analyzed by MALDI-TOF MS using the simplified method. Twenty-eight out of 32 of the urediniospore samples generated spectra, which were analyzed using principal-component analysis as shown in **Figure 3**. As is evident from **Figure 3**, the Indian and Pakistani strains of Himalayan balsam rust can clearly be differentiated by MALDI-TOF MS analysis of urediniospores. The India-strain samples form a fairly tight PCA cluster but the samples originating from Pakistan form a more diffuse cluster. The discrimination between strains is unlikely to be due to material from host plants because spectral comparisons show no similarity between the rust spectra and those of the host plant *Impatiens glandulifera* (data not shown). Example spectra for the India-strain Himalayan balsam rust (**Fig. 4A**) and Pakistan-strain Himalayan balsam rust (**Fig. 4B**) are also shown.

### Closely-related *Crassula* spp. *vs*. regional biotypes of *Crassula helmsii*

Six reference leaves and six “blind-test” leaves from: *Crassula aquatica*; *Crassula helmsii*, UK; *Crassula helmsii*, Victoria, Australia; *Crassula helmsii*, Flinder’s Island, Australia; and *Crassula helmsii*, Belgium were used to construct database mass spectra, which were then used to identify the unknown “blind-test” samples. The resulting identification data are shown in **Table S2**. As can be seen in **Table S2**, in the majority of cases, the best matches and second-best matches are all congruent. In two cases, however, the best match and second-best match are not congruent. This reflects the very high similarity between the UK and Belgian biotypes of *Crassula helmsii* and reduces the identification confidence of blind-test samples 13 and 23. Using the best matches gives the following identifications: *Crassula helmsii*, UK (samples 6, 8, 18, 23, and 29); *Crassula helmsii*, Victoria, Australia (samples 3, 9, 14, 19, 24, and 25); *Crassula helmsii*, Flinder’s Island, Australia (samples 2, 10, 11, 17, 22, and 27); *Crassula helmsii*, Belgium (samples 5, 12, 13, 15, 20, 21, and 28); and *Crassula aquatica* (samples 1, 4, 7, 16, 26, and 30). There are therefore seven samples identified in this manner as *Crassula helmsii* from Belgium and five samples as *Crassula helmsii* from the UK. With the additional information that there were six samples in each category, one would be able to reassign sample 13 as *Crassula helmsii* from the UK but this would not be possible in routine identification without this additional information. All of the above identifications were therefore correct upon unblinding the “blind-test” samples except that blind-test sample 13 was misassigned as *Crassula helmsii* from Belgium rather than *Crassula helmsii* from the UK.

### Rubbervine introduced into Australia *vs*. Brazil

Pressed-and-dried samples of Australian rubbervine (*Cryptostegia grandiflora*) and Brazilian rubbervine (possibly *Cryptostegia madagascariensis* or a *Cryptostegia* hybrid) were analyzed using the simplified method, with multiple leaves and multiple plants from each strain. Reference samples were used to construct database mass spectra, which were then used to identify the unknown “blind-test” samples. The resulting identification data are shown in **Table S3**, which were 100% correct upon unblinding. Clear discrimination between Australian and Brazilian rubbervine is therefore possible using MALDI-TOF MS analysis of pressed-and-dried leaf material and, moreover, in all cases, the top eight matches were congruent in terms of Australian or Brazilian rubbervine (data not shown).

### Analysis of fall armyworm and stem-borer samples stored in 70% ethanol

**Figure 5.**
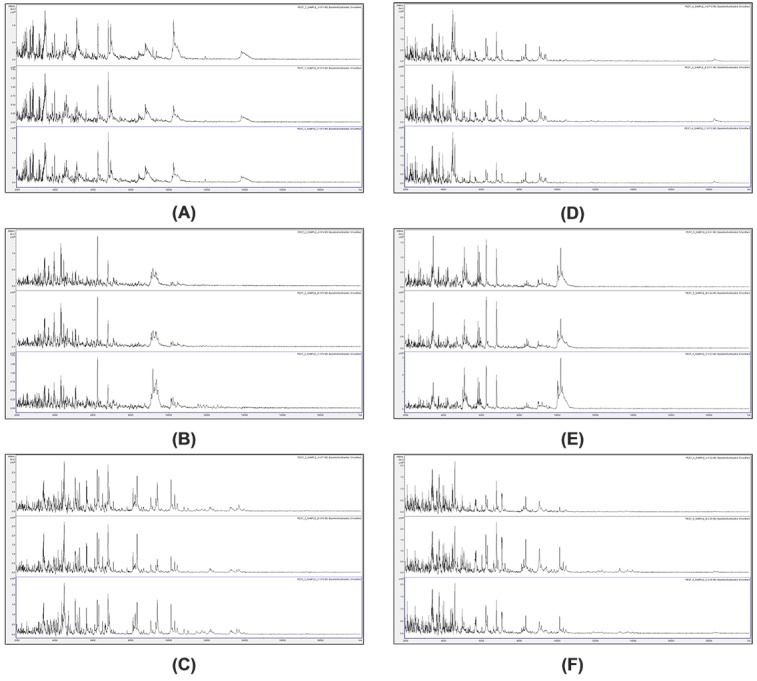
Example mass spectra for insect samples PEST 1 to PEST 6. Example mass spectra (baseline-subtracted, smoothed, *y*-axis-autoscaled, and covering the mass range 2 kDa to 20 kDa) for the A (top), B (middle), and C (bottom) triplicate samples of PEST 1 (A), PEST 2 (B), PEST 3 (C), PEST 4 (D), PEST 5 (E), and PEST 6 (F).

**Figure 6.**
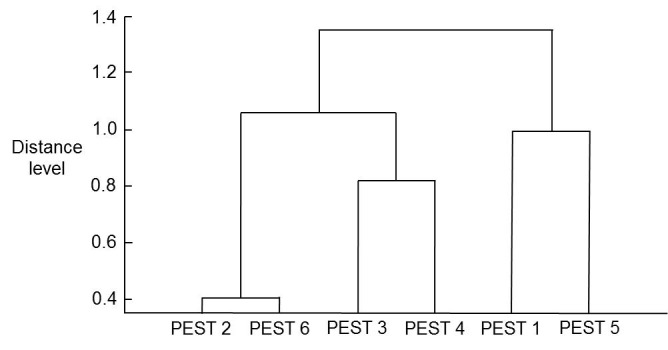
PCA dendrogram for the insect samples PEST 1 to PEST 6. PCA dendrogram derived from the mass spectra for the insect samples PEST 1 to PEST 6 (figure redrawn from original Bruker image). Spectra were analyzed by PCA using the Bruker Online Client software to give the dendrogram. PCA analysis was unsupervised, and all peaks were weighted equally.

This preliminary study was performed blind with six insect samples (PEST 1-6) archived in 70% (v/v) ethanol for up to nine months. Triplicate sample-preparations were carried out from each insect and the mass spectra for the A (top), B (middle), and C (bottom) samples of each insect are shown in **Figure 5**. Principal-component analysis was then carried out from the A samples, resulting in the dendrogram shown in **Figure 6**. The unblinded sample identities are PEST 1 (*Chilo partellus*, Malawi), PEST 2 (*Spodoptera frugiperda* type 1 (rice), Malawi), PEST 3 (*Spodoptera frugiperda* type 2 (corn), Zambia), PEST 4 (*Spodoptera frugiperda* type 1 (rice), Zambia), PEST 5 (*Busseola fusca*, Malawi), and PEST 6 (*Spodoptera frugiperda* type 1 (rice), Rwanda). Mass spectra can therefore clearly be obtained from old samples stored in 70% (v/v) ethanol using this method. Spectral duplication between triplicates is good, and blind testing was able to discriminate between fall armyworm and stem-borer samples. The dendrogram is as expected except for the *Spodoptera frugiperda* type 1 (Zambia) sample (PEST 4), which appears on MALDI-TOF MS analysis to be more closely related to *Spodoptera frugiperda* type 2 (Zambia) sample (PEST 3) than to the *Spodoptera frugiperda* type 1 (Malawi) sample (PEST 2) and the *Spodoptera frugiperda* type 1 (Rwanda) sample (PEST 6), suggesting that the region of origin might determine the MALDI-TOF MS spectral profile more than the distinction between type 1 (rice) and type 2 (corn) based upon mitochondrial DNA barcoding.

### Analysis of old dried insect specimens

**Figure 7.**
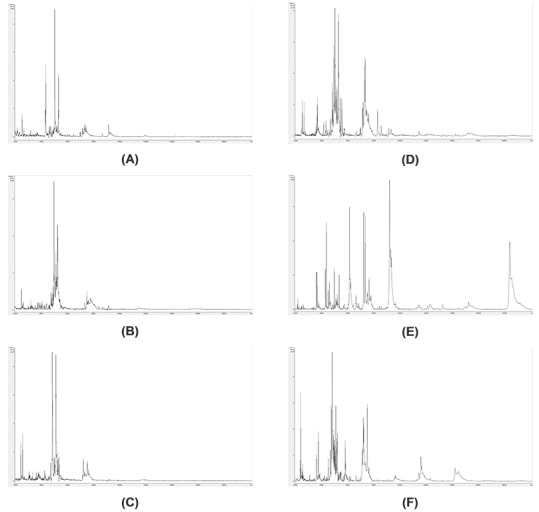
Example mass spectra for old dried insect specimens. Example mass spectra (baseline-subtracted, smoothed, *y*-axis-autoscaled, and covering the mass range 2 kDa to 20 kDa) for *Laothoe populi* (1966) (A), *Smerinthus ocellata* (1966) (B), *Deilephila elpenor* (1966) (C), *Laothoe populi* (2012) (D), *Smerinthus ocellata* (2017) (E), and *Deilephila elpenor* (2013) (F).

**Figure 8.**
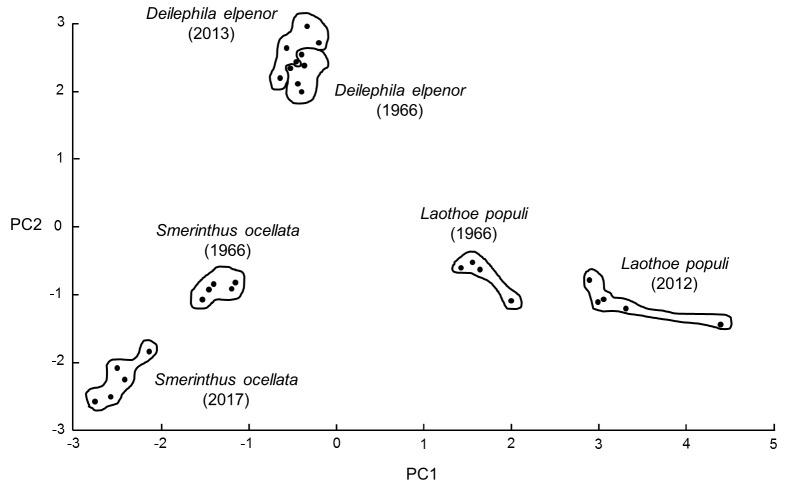
PCA ordination plot for old dried insect specimens. PCA ordination plot for mass spectra generated by samples of *Laothoe populi* (1966), *Smerinthus ocellata* (1966), *Deilephila elpenor* (1966), *Laothoe populi* (2012), *Smerinthus ocellata* (2017), and *Deilephila elpenor* (2013) (figure redrawn from original Bruker image). Spectra were analyzed by PCA using the Bruker Online Client software, with the components of the first principal component vector shown in the *X* direction and the components of the orthogonal second principal component vector shown in the *Y* direction to give the ordination plot. PCA analysis was unsupervised, and all peaks were weighted equally.

Adult insect legs from the Sphingidae species indicated were stored dry since sampling on the dates indicated in **Table S4**. Representative mass spectra obtained using the simplified method are shown in **Figure 7**. From the information after unblinding, it is clear that the better-quality spectra are those of more-recent origin (stored dry for up to five years) and that the 1966 samples (stored dry for 51 years) give the poorest-quality spectra. A PCA ordination plot for the 29 samples, annotated after unblinding, is given in **Figure 8**. The 2012–2017 (“new”) dried specimens gave reasonable-quality to high-quality spectra unlike the 1966 (“old”) dried specimens, which gave spectra with significantly-reduced peak richness. Identification of new test samples using new-sample database entries gave very high-confidence Bruker scores (average value 2.431), identification of old test samples using new-sample database entries gave low-confidence Bruker scores (average value 1.615), identification of old test samples using old-sample database entries gave high-confidence Bruker scores (average value 2.137), and identification of new test samples using old-sample database entries gave low-confidence Bruker scores (average value 1.625), where a Bruker score of between 2.300 and 3.000 indicates highly-probable species-level identification, a score between 2.000 and 2.299 indicates secure genus-level identification and probable species-level identification, a score of between 1.700 and 1.999 indicates probable genus-level identification, and a score between 0.000 and 1.699 indicates no reliable identification (**Table S5**).

## DISCUSSION

A highly-simplified and inexpensive method for MALDI-TOF MS sample-preparation has been developed with broad utility to microorganisms, plants, and insects. The optimal reagent formulation for the simplified method is HCCA matrix close to saturation in 60%–65% (v/v) acetonitrile in water containing 2.5% (v/v) TFA. In this paper, we have used this simplified method to differentiate between strains for a representative set of aflatoxin-producing and aflatoxin-non-producing strains of *Aspergillus* fungi, to differentiate between Indian and Pakistani strains of Himalayan balsam rust, to differentiate between closely-related *Crassula spp.* and regional biotypes of *Crassula helmsii*, to differentiate between rubbervine introduced into Australia and Brazil, for the analysis of fall armyworm and stem-borer samples stored in 70% (v/v) ethanol, and for the analysis of old dried insect specimens.

The development of this simplified method was guided by the following thought process. Given the method requirements, acidified acetonitrile was chosen as the solvent basis for the desired reagent formulation, with the aim of causing cell lysis by disruption of the membrane-stabilizing hydrophobic effect using high concentrations of acetonitrile and selective ribosomal-protein solubilization through low pH. TFA was chosen for acidification because it is a significantly stronger acid than formic acid. This means that comparable proton concentrations can be obtained from much lower concentrations of acid, thereby dramatically reducing the amount of odorous material evaporating from the reagent during use. In methods reported to date, after protein extraction, the acidic supernatant from formic acid treatment is dried down onto the sample-plate prior to overlay with matrix solution. Once dried down onto solid surfaces, many proteins often re-solubilize inefficiently. We therefore decided to premix the proteins and matrix and then dry these down together. In order to make this economically feasible, inexpensive matrix is necessary and so we decided to employ less-expensive grades of HCCA, with ≥ 98% pure (TLC-grade) C2020-10G matrix supplied by Sigma being, for example, around 500 times cheaper per gram than many 99%-pure “MALDI-grade” reagents. In direct-transfer protocols, highly-purified matrix is eventually mixed with a crude cell lysate so we reasoned that the importance of small differences in reagent purity is perhaps overstated given our intended usage.

By comparison, existing direct-transfer protocols are rapid to perform but they are not well suited to the transfer of some types of fungal biomass, especially powdery forms of mycelium and spores. The method described in this paper is equally rapid to perform but has the advantage that it is better suited to the transfer of such forms of biomass. By contrast, full-extraction protocols are able to handle many more types of fungal biomass but they are vulnerable to loss of biomass after the first centrifugation step when small amounts of material are used (which can be particularly problematical for fungal species and strains that predominantly grow invasively into the agar substrate when cultured). The current method suffers no such vulnerability to small amounts of biomass and is significantly quicker to perform. In addition, because it does not require centrifugation, it is less constrained in terms of throughput and can more readily be employed in resource-poor settings.

In terms of breadth of applicability, neither direct-transfer protocols nor full-extraction protocols are particularly suited to the analysis of plant or insect material, for which the method described in this paper is both convenient and highly effective. In particular, this method avoids the use of formic acid, which is strongly odorous and is used at very high molar concentrations (often 19 M) in MALDI-TOF MS sample-preparation methods. With its simplicity and effectiveness, the method described in this paper facilitates subtle differentiation between plant samples and between insect samples and, in many cases, at higher resolution than species level, as demonstrated above. The ability to discriminate rapidly and inexpensively between plant or insect populations at this resolution has enormous potential in terms of monitoring the spread of difficult-to-identify or genetically-diverse insect pests (such as fall armyworm) and increasing both the efficiency and the efficacy of biological-control approaches to the management of invasive weeds (such as Himalayan balsam) and insect pests.

In terms of limitations, whilst we have, so far, found the method to be generally applicable to plants, insects, bacteria, and many fungi, for yeasts, we have encountered cases (*e.g.*, *Saccharomyces cerevisiae*) where the chosen reagent formulation is incapable of lysing the cell wall. For *Saccharomyces cerevisiae*, we have found that pre-treatment of the yeast cells in 1/10^th^ volume of 70% (v/v) formic acid prior to adding the acetonitrile, TFA, and HCCA mixture can be an effective solution to this problem. As with direct-transfer protocols, the simplified method that we have developed does not include a pre-treatment of the sample material with 70% (v/v) ethanol, which is generally considered to be an effective microbicide. As it is not certain that there will be complete biological inactivation of any microorganisms in the crude lysates generated by this method, biological containment and other precautionary measures employed must be at an appropriate level for the pathogenicity or potential pathogenicity of any microorganisms used with any methods described in this paper.

For the analysis of old dried insect samples, the method used here cannot be used reliably to associate relatively new (< 5 years) insect samples with relatively old reference samples (51 years). However, both our trials suggest that relatively new insect samples can be reliably associated with other relatively new samples of the same species. This should be particularly useful for the rapid processing of large numbers of samples in a focused programme (*e.g.*, monitoring a particular pest and its introduced biological control agent, where confusingly similar species exist).

## Supplementary Material

Supplementary information **Table S1.** Spectral similarity analysis for Aspergillus samples.
**Table S2.** Identification results for Crassula samples.
**Table S3.** Identification results for rubbervine samples.
**Table S4.** Old (1960s) and new (2010s) dried Sphingidae sample details.
**Table S5.** Identification results for Sphingidae samples.
Supplementary information of this article can be found online at http://www.jbmethods.org/jbm/rt/suppFiles/261.
